# Are life history events of a northern breeding population of Cooper's Hawks influenced by changing climate?

**DOI:** 10.1002/ece3.2619

**Published:** 2016-12-20

**Authors:** Robert N. Rosenfield, Madeline G. Hardin, John Bielefeldt, Edward R. Keyel

**Affiliations:** ^1^ Department of BiologyUniversity of WisconsinStevens PointWIUSA

**Keywords:** *Accipiter cooperii*, adaptive response, climate change, clutch size, Cooper's Hawk, egg‐hatching dates, shift in reproductive timing

## Abstract

Numerous studies have demonstrated earlier timing of spring migration and egg‐laying in small passerines, but documentation of such responses to recent climate change in the life histories of higher trophic feeding birds such as raptors is relatively scarce. Raptors may be particularly susceptible to possible adverse effects of climate change due to their longer generation turnover times and lower reproductive capacity, which could lead to population declines because of an inability to match reproductive timing with optimal brood rearing conditions. Conversely adaptively favorable outcomes due to the influence of changing climate may occur. In general, birds that seasonally nest earlier typically have higher reproductive output compared to conspecifics that nest later in the season. Given the strong seasonal decline in reproductive output, and the heritability of nesting phenology, it is possible that nesting seasons would (adaptively) advance over time. Recent climate warming may release prior ecological constraints on birds that depend on food availability at the time of egg production, as do various raptors including Cooper's Hawks (*Accipiter cooperii*). Under this scenario, productivity, especially clutch size, might increase because it is likely that this reproductive demographic may be the most immediate response to the earlier seasonal presence of food resources. We demonstrated a statistically significant shift of about 4–5 days to an earlier timing of egg‐hatching in spring across 36 years during 1980–2015 for a partially migratory population of Cooper's Hawks in Wisconsin, United States, which is consistent with a recent study that showed that Cooper's Hawks had advanced their timing of spring migration during 1979–2012. Both studies occurred in the Great Lakes region, an area that compared to global averages is experiencing earlier and increased warming particularly in the spring in Wisconsin. The nesting period did not lengthen. We suggest that the gradual shift of six consecutive generations of hawks was likely in response to recent climate change or warming. We did not detect any long‐term temporal change in average clutch or brood sizes. However, such indices of reproduction are among the highest known for the species and thus may be at their physio‐ecological maximum for this population. Our study population appears to show resilience to and does not appear to be adversely influenced by the recent rate of changing climate at this time.

## 1 Introduction

Much evidence suggests that recent climate change has altered life history events of numerous species of birds (e.g., Brommer, 2004; Jenni & Kery, 2003; Wood & Kellermann, 2015). Studies particularly demonstrate earlier timing of spring migration and egg‐laying in birds throughout the Northern Hemisphere, although these findings are strongly biased toward research on small passerines (Dunn, 2004; Lehikoinen, Saurola, Byholm, Lindén, & Valkama, 2010; Nielsen & Møller, 2006). There are relatively few studies that have investigated the possible effects of climate change on higher‐level avian consumers, including predatory hawks (Clutton‐Brock & Sheldon, 2010; Lehikoinen et al., 2010, 2013). However, some recent studies on raptors have shown earlier spring migration (Jaffré et al., 2013; Lehikoinen et al., 2010; Sullivan, Flaspohler, Froese, & Ford, 2016), earlier hatching dates (Lehikoinen et al., 2013), and shifts to earlier (Filippi‐Codaccioni, Moussus, Urcun, & Jiguet, 2010) and later timing of autumnal migrants (Rosenfield, Lamers, Evans, Evans, & Cava, 2011; Van Buskirk, 2012), with such seasonal shifts being consistent with compensatory response to global warming (Sullivan et al., 2016).

Sullivan et al. (2016) recently demonstrated phenological shifts to earlier spring migration of several raptor species, including Cooper's Hawks during 1979–2012 in the Great Lakes region of North America, and they indicated that such shifts were consistent with decadal climatic oscillations and global climate change. Such shifts could likely lead to earlier arrival times at breeding territories, which in turn could lead to earlier breeding schedules, a lengthened nesting period, and possibly increased productivity (perhaps an adaptive response sensu Lehikoinen et al., 2010; Fontaine, Stutzman, & Gannes, 2015). Alternatively, it is possible that changes in timing of migration could produce maladapted individuals given a possible decoupling between migration (and breeding) schedule of hawks and the temporal variation in available prey (Lehikoinen et al., 2009; Rosenfield et al., 2011). However, the possible consequences on the aforementioned life history events are unknown for the raptors in the Great Lakes region studied by Sullivan et al. (2016). Indeed, Sullivan et al. (2016) indicated concern for the viability of these species' regional populations given in part the lack of data to indicate whether behavioral responses are sufficient to maintain adequate productivity, compounded with the fact that raptors tend to exhibit relatively low reproductive capacities. This concern is significantly aggravated by the fact that Sullivan et al. (2016) do not know the specific destinations and/or breeding locales of the individuals of the various species counted and thus where population data for each species could be gathered to potentially address their concern. Moreover, Sullivan et al. (2016) noted that certain life history attributes (e.g., longer generation turnover times) appear to constrain responsiveness to changing climate, which could lead to asynchrony in phenological events across trophic levels and cause population declines. Mismatches between reproductive timing and food availability are expected to become more severe with increasing trophic level (Both, Van Asch, Bijlsma, Van Den Burg, & Visser, 2009; Visser, Both, & Lambrechts, 2004).

Lehikoinen et al. (2010) demonstrated earlier timing of spring arrival dates and hatching dates in the migratory Eurasian Sparrowhawk (*Accipiter nisus*) in Finland and attributed these results to climate change (increased warming in the spring) in their study areas across 29 years (1979–2007). This small raptor is a specialized predator of passerines and perhaps responded to the earlier arrival of their migratory prey (Lehikoinen et al., 2010). They suggested that this temporal dynamic provided improved hunting conditions and allowed sparrowhawks to gain resources for breeding more easily during the preincubation period and, in turn, could advance timing of breeding and increase productivity. Notably, they documented increased mean brood sizes in this raptor but claimed that this result was likely due to reduced exposure to organochlorine pollutants. However, they indicated that they could not exclude the possibility that an earlier onset of breeding had caused increased production.

Recent climate change may release prior ecological constraints on birds that depend on resource availability at the time of egg production (e.g., Cooper's Hawks [*Accipiter cooperii*]; Drent, Fox, & Stahl, 2006; Gienapp & Visser, 2006; Rosenfield, Bielefeldt, & Cary, 1991; Rosenfield, Sonsthagen, Stout, & Talbot, 2015). Cooper's Hawks in our Wisconsin population and other birds that nest earlier in the season typically have higher reproductive output of eggs and/or offspring compared to conspecifics that nest later in the season (Perrins, 1970; Newton, 1979; Rosenfield & Bielefeldt, 1999; R.N. Rosenfield, unpubl. data). It is possible that nesting season would advance over time as a consequence of an adaptive response by breeding birds especially given the strong seasonal decline in reproductive output, and the heritability of nesting phenology (van Noordwijk, van Balen, & Scharloo, 1981; Van der Jeugd & McCleery, 2002). Thus, it is possible that an advanced timing of nesting could result in increased productivity, as was shown for *Ficedula* flycatchers in Europe in which earlier advancement of laying prompted by climate change (i.e., increased spring warming) resulted in increased clutch sizes (Both et al., 2004).

Clutch size is perhaps the most central index to avian reproduction as it exhibits a strong correlation with age at maturity, and offspring and adult survival in birds (Jetz, Sekercioglu, & Bohning‐Gaese, 2008; Ricklefs, 2000). Seasonality of resources is globally the predominant driver of clutch variation in birds across geographic gradients (Jetz et al., 2008). We contend it would have been particularly revealing if Lehikoinen et al. (2010) had found that clutch sizes had increased over the study period (clutch counts were not presented) when hatching dates advanced because it is likely that this reproductive demographic may be the most temporally proximate or immediate response to the earlier arrival of prey by sparrowhawks. We note that higher mean clutch counts, but not mean brood counts, occur in years with earlier hatch dates in our Wisconsin populations of Cooper's Hawks (Rosenfield & Bielefeldt, 1999; and see Section “2”). Clutch size also indexes reproductive potential, life history trade‐offs pertinent to migratory behavior in birds including possibly Cooper's Hawks (Meiri & Yom‐Tov, 2004; Rosenfield et al., 2007). To our knowledge, there are no studies of higher trophic predatory birds in which variation in clutch size was investigated as a potential demographic response to climate change.

We conducted long‐term nesting studies of a partially migratory population of Cooper's Hawks in Wisconsin, which population is near the northern extent of this species' continental range in the Great Lakes region (Rosenfield & Bielefeldt, 1993; Bielefeldt, Rosenfield, Stout, & Vos, 1998; R.N. Rosenfield, unpubl. data). Some adult breeding and hatching‐year Wisconsin Cooper's Hawks overwinter in the state, and individuals of both age cohorts migrate and overwinter as far south as Central America (R.N. Rosenfield, unpubl. data). We note that autumnal migrant Cooper's Hawks along the western shore of Great Lakes' Lake Michigan have been documented breeding in our study areas, and tallies of such migrant Cooper's Hawks have been consistent with breeding population trends for Cooper's Hawks in our study areas (Rosenfield, Bielefeldt, Booms, Cava, & Bozek, 2013; Rosenfield, Hardin, Bielefeldt, & Anderson, 2016).

We note that Sullivan et al. (2016) reported Cooper's Hawk to have advanced its spring migration in our study region during the study years included herein and that changes to higher average temperatures from 1950 to 2009 in the Great Lakes region exceed global averages (Hayhoe, Vandorn, Croley, Schlegal, & Wuebbles, 2010). Wisconsin is among the 10 fastest warming states in the United States (Tebaldi, Adams‐Smith, & Heller, 2012), particularly regarding increased spring temperatures documented in our study areas (Kucharik, Serbin, Vavrus, Hopkins, & Motew, 2010; Zhao & Schwartz, 2003). Further, there is strong evidence of a general and gradual trend of advanced spring plant phenology and delays in the onset of fall throughout the Great Lakes region including Wisconsin throughout our study years (Bradley, Leopold, Ross, & Huffaker, 1999; Ewert, Hall, Smith, & Rodewald, 2015; Kucharik et al., 2010). Several species of migratory birds preyed upon by Cooper's Hawks in our study sites (e.g., American Robin [*Turdus migratorius*], red‐winged blackbird [*Agelaius phoeniceus*]; Bielefeldt, Rosenfield, & Papp, 1992; R.N. Rosenfield, unpubl. data) exhibited a significantly gradual and earlier spring arrival to Wisconsin during our study years (Bradley et al., 1999). The advanced timing of spring migration of these birds was linked to earlier and higher spring temperatures due to climate change (Bradley et al., 1999). Notably, one of these species, the American Robin, is the most commonly detected prey item in spring during the preincubation period at Cooper's Hawk nest sites on our study areas throughout all study years (Bielefeldt et al., 1992; R.N. Rosenfield, unpubl. data), and thus, likely an important resource for accumulation of body reserves requisite for egg production (Rosenfield & Bielefeldt, 1999).

We use a 36‐year data set involving six consecutive generations (estimated at ~6 years per generation (Rosenfield, Bielefeldt, Affledt, & Beckmann, 1995)) of Cooper's Hawks during 1980–2015 to investigate whether our study population has gradually advanced its egg‐laying period during spring in accord with documented gradual spring warming in Wisconsin during our study years. We also assessed whether average clutch and/or brood sizes have increased (sensu Lehikoinen et al., 2010) such that potential changes in all three life history events may be consistent (or adaptive) with recent climate warming in Wisconsin (Kucharik et al., 2010) for this primarily bird‐catching hawk whose diet includes migratory birds (Bielefeldt et al., 1992, 1998; Rosenfield et al., 2010, R.N. Rosenfield unpubl. data).

## 2 Methods

### 2.1 Study areas

We studied breeding Cooper's Hawks during 1980–2015 at two principal areas in central and southeastern parts of Wisconsin as described by Rosenfield et al. (1995, 2010) and Rosenfield and Bielefeldt (1996). Our central Wisconsin area principally included the rural environs of Portage County and within the same county the abutting municipalities of Stevens Point, Whiting, and Plover, with a predominately urban human population about 38,000, and a human density about 600/km^2^ (US Department of Commerce, 2000). Our southeastern Wisconsin area principally involved rural environs of the Kettle Moraine State Forest, South Unit. These study sites were chosen without preconceptions about their suitability for nesting Cooper's Hawks (Bielefeldt et al., 1998). We note that we have been unable to show in our multidecadal studies that habitat (i.e., rural or urban, conifer plantation or nonplantation, presumptive site quality as indexed by consistency of nesting area use, and high breeding density) is related to size of clutch or brood counts, nesting phenology, annual adult survival, and production of recruits, or fitness in Cooper's Hawks in Wisconsin (Rosenfield & Bielefeldt, 1999; Rosenfield, Bielefeldt, Sonsthagen, & Booms, 2000; Rosenfield, Bielefeldt, Rosenfield, Booms, & Bozek, 2009; Rosenfield et al., 2015; Rosenfield, Stout et al., 2015; Rosenfield, Hardin et al., 2016, R.N. Rosenfield, unpubl. data).

### 2.2 Field procedures

Each year, we found most nests (>90%) before egg‐laying by listening for dawn vocalizations (Rosenfield & Bielefeldt, 1991) or by searching for partially constructed nests during the preincubation stage, about mid‐March through late April (Bielefeldt et al., 1998). We are thus able to examine productivity without adjusting for the biases that might have resulted from excluding breeding attempts that failed prior to discovery (Rosenfield et al., 2000). Further descriptions of our study design, study areas, and nest finding techniques can be found in Rosenfield and Bielefeldt (1991, 1996).

We made at least two visits to nests to assess reproduction. One visit included climbing to nests during the mid‐incubation period (about mid‐May) to obtain completed clutch counts and another climb about mid‐June when nestlings were 16–19 days of age, or about 70% of fledgling age, to ascertain brood size, age, and band young at successful nests (Rosenfield & Bielefeldt, 2006; Rosenfield, Grier, & Fyfe, 2007). This schedule avoided the criterion of 80% of fledgling age suggested for other raptor species, an age at which visits could result in premature fledging of some nestlings and/or inaccurate brood counts of young (Rosenfield, Grier et al., 2007; Rosenfield, Grier, & Fyfe, 2007). Brood counts herein are from successful nests in which at least one young reached 16–19 days of age (Rosenfield et al., 2013). Hatching dates per nest were determined by backdating from estimated nestling ages of the oldest chick based on plumage development of known‐age birds (Meng & Rosenfield, 1988; Bielefeldt et al., 1998, R.N. Rosenfield unpubl. data). Estimated hatching dates were based only on young aged by RNR, who aged >95% of all bandable young each year across all study years.

### 2.3 Data analyses

We used simple linear regression to assess how Julian date for the 50th percentile (i.e., median) of the of the total number of nests with estimated hatching dates in each of 36 study years changed across time (i.e., to ascertain whether a statistically significant shift in hatching date per year had occurred following procedures in Rosenfield et al. (2011)). We used linear regression because it is often used to detect possible changes of timing of events in migratory birds due to climate change (e.g., Wood & Kellermann, 2015); linear regression was also used to investigate the possible effects of climate warming on timing of abiotic events and phenologies of 55 different species of plants and birds (including songbirds preyed upon by Cooper's Hawks) in Wisconsin during our study years (Bradley et al., 1999; Ewert et al., 2015; Serbin & Kucharik, 2009). Invocation of similar analytical techniques by different researchers studying similar phenomenon facilitates a tenable discussion of results across studies (Whitlock & Schluter, 2009). We used the median because it was less sensitive to the effects of data outliers that could skew results (see Rosenfield et al., 2011).

**Figure 1:**
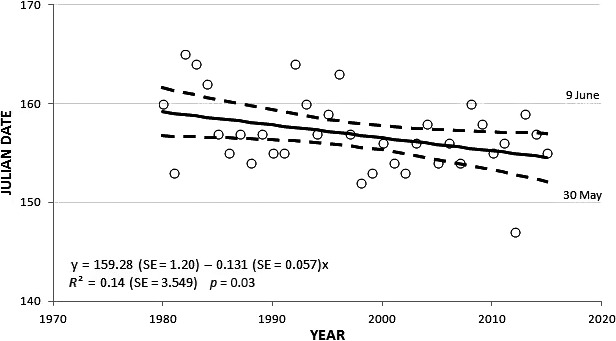
Relationship between Julian dates of the 50th percentile of the egg‐hatching dates (open circles) of Cooper's Hawks and year of breeding in Wisconsin, 1980–2015. Regression line is bounded by 95% confidence interval. Calendar dates are included for descriptive reference

We also used Julian dates for four percentiles (25, 50, 75, and 100) of hatch dates of nests in each year to describe the extent of the shift in days of the hatching dates across years following Rosenfield et al. (2011). We calculated the difference in number of Julian dates (days) for each percentile in each year for 1981–2015 relative to Julian dates for the respective percentiles in 1980, the first year of the study. Julian dates earlier and later than those in the respective percentiles for 1980 were assigned negative and positive values, respectively. We used the average (*SE*) of those differences to enumerate the approximate shift in days of hatching date for each respective percentile since 1980. We chose 1980 as the comparative year to demonstrate the extent of the shift in hatching dates because it was representative of the earlier timing of hatching dates at the outset of the study (Figure 1). The tenability of using 1980 as an earlier and comparative temporal base line is underscored by the fact that the median Julian hatch date of 160 in 1980 (Table 1) was same as the average of median Julian hatch dates for the first (1980–1985) of six study generations of Cooper's Hawks. We also report the extent of the shift in hatching dates for all combined percentiles, 1981–2015. We subtracted the difference between the 100th and 25th percentiles to determine whether the length of the nesting period had changed between 1980 and 1981–2015.

**Table 1:** Timing of egg‐hatching dates for Cooper's Hawks in Wisconsin, 1980–2015. Julian dates for 1980 indicate when the specified proportion of the egg‐hatching dates for the total number of nests was attained; Julian dates for other years are rounded approximations of percentile attainment based on mean values (in parentheses [*SE*]) of shift in days relative to Julian dates in 1980. Calendar dates are provided for descriptive reference

Percentile	1980	1981–2015	Mean shift in days from 1980
25	157; 6 June	152; 1 June	−4.94 (0.69)
50	160; 9 June	157; 6 June	−3.06 (0.64)
75	168; 17 June	161; 10 June	−5.94 (0.79)
100	176; 25 June	172; 21 June	−2.86 (0.91)

We also used simple linear regression to assess how mean number of eggs/nest per year and mean number of nestlings/nest per year varied to ascertain whether a temporal trend in these life history events had occurred across all study years. Mean values are conventionally used by raptor researchers in both descriptive statistics and as metrics in inferential decisions regarding reproductive output, including studies regarding possible effects of climate change on productivity (e.g., Lehikoinen et al., 2010; Rosenfield & Bielefeldt, 1993; Rosenfield, Grier et al., 2007). We note in nontime series analyses that higher mean clutch counts occur in years of earlier hatch dates (*y* = 8.19 [*SE* = 1.74] − 0.03 [*SE* = 0.01]*x*,* R* ^2^ = 0.13 [*SE* = 0.25], *p* = .03), but mean brood counts are not related to hatch dates (*y* = 6.25 [*SE* = 1.71]  − 0.02 [*SE* = 0.01]*x*,* R* ^2^ = 0.06 [*SE* = 0.24], *p *=* *.14).

Statistical procedures followed Whitlock and Schluter (2009). We calculated test statistics and probability values using SYSTAT 10 for Windows (SPSS, 2000). Statistical significance was accepted at *p *≤* *.05. We used the Durbin‐Watson *D* statistic to assess for possible autocorrelation in our time series regressions; the *D* statistic ranges from 0 to 4, with scores at or near 2 indicating no autocorrelation. *D* values of 1.8 for regressions of median egg‐hatching dates and mean clutch counts by year, and a *D* statistic of 2 for regressions of mean brood counts indicated no evidence of autocorrelation in our three time series analyses (all *p*s > 0.05).

## 3 Results

We obtained egg‐hatching dates for 691 Cooper's Hawk nests in Wisconsin across 36 years, 1980–2015 (range of total nests with hatching dates per year was 8–33, with means and medians of 20 nests/year). There was a weak but statistically significant gradual decrease in Julian dates for the 50th percentile of hatching dates per year across all study years (slope = −0.131, *SE* = 0.057, *p *=* *.03); thus, greater proportions of nests had earlier egg‐hatching dates (resulting in an estimated earlier hatch timing of 0.13 days per year; Figure 1). Of 140 total Julian hatch dates for the first through fourth percentiles, 109 (78%) registered an earlier day of percentile attainment during 1981–2015 versus 1980. The extent of the shift in egg‐hatching was about 3–6 days earlier compared to 1980 in the first through fourth percentiles of Julian hatch dates during 1981–2015 (Table 1). The shift was on average 4.2 (*SE* = 0.39; median = 5) days earlier for all 144 percentiles combined across 36 years. The small range of 3–6 days to earlier hatching among all four sets of comparative percentiles suggests that the length of the nesting period remained similar across all study years; the subtracted differences between the 100th and 25th percentile Julian dates in 1980 (176 minus 157) and in 1981–2015 (172 minus 152) were 19 and 20 days, respectively (Table 1).

**Figure 2:**
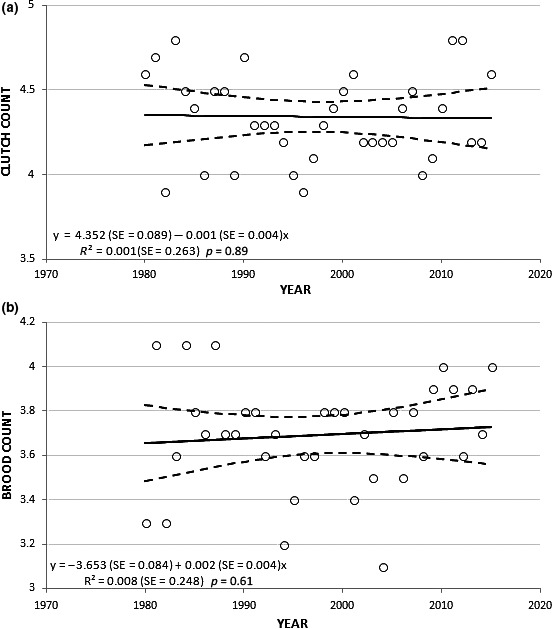
Relationship between mean clutch counts (a) and mean brood counts (b) of Cooper's Hawks and year of breeding in Wisconsin, 1980–2015. Regression lines for clutch and brood counts are bounded by 95% confidence intervals

We obtained clutch and brood counts for 613 and 740 nests, respectively, across all study years, 1980–2015. Mean clutch sizes ranged from 3.9 to 4.8 eggs/year, with an overall average of 4.3 eggs/year (*SE* = 0.03; median = 4); mean brood sizes ranged from 3.1 to 4.1 nestlings/year and an overall average of 3.7 young/year (*SE* = 0.04; median = 4) across all study years (Figure 2). We did not detect a statistically significant trend in mean clutch (slope = −0.001, *SE* = 0.004, *p* = .89) and mean brood sizes/year (slope = 0.002, *SE* = 0.004, *p* = .61) across 36 years in Wisconsin (Figure 2). We conclude that the gradual change to an earlier nesting phenology did not appear to influence average clutch or brood counts in our study population of Wisconsin Cooper's Hawks.

## 4 Discussion

Although numerous studies have demonstrated earlier timing of spring migration and egg‐laying in small passerines (Wood & Kellermann, 2015), documentation of such responses to recent climate change in the life histories of secondary consumer birds such as raptors is scarce (Lehikoinen et al., 2009, 2010) . We are unaware of any study that investigated potential variation or increase in clutch counts in raptors as a possible adaptive response to changing climate. Sullivan et al. (2016) recently demonstrated earlier timing of spring migration for several raptors, including Cooper's Hawks, in the Great Lakes region, and attributed such to recent climate warming, but they did not provide any data on possible breeding responses by these species in this region due to their advanced timing of migration. Herein, we demonstrated a statistically significant, gradual shift to earlier timing of egg‐hatching across 36 years (1980–2015) for a partially migratory population of Cooper's Hawks in Wisconsin during the same study period and within the same region investigated by Sullivan et al. (2016). We suggest that the gradual shift involving six consecutive generations of Cooper's Hawks to earlier nesting phenology in our study population is in accord with the finding of Sullivan et al. (2016) for earlier spring migration timing (due to climate warming) for this species in the Great Lakes region in our study years. We note that the shift in timing of egg‐laying exhibits other consistencies with responses of species to recent changing climate and gradual warming in this region including Wisconsin (Bradley et al., 1999; Ewert et al., 2015). For example, the gradual shift of 0.31 days/year in spring to earlier egg‐laying in Wisconsin Cooper's Hawks is similar to a gradual and average of 0.21 days/year of earlier timing of collective spring phenologies for 55 species of Wisconsin plants and birds during our study years (these birds were predominately migratory songbirds whose earlier arrival may trigger egg‐laying in Cooper's Hawks, see below; Bradley et al., 1999). We note that a gradual pace of response to climate warming is common for many species (including birds), both globally and in Wisconsin (Bradley et al., 1999; Kucharik et al., 2010; Stanley, 1988; Wood & Kellermann, 2015). We also highlight that the shift to earlier hatching of Cooper's Hawks occurred seasonally during spring, which, along with winter, are the seasons exhibiting the greatest warming in Wisconsin on our study areas during our study years (Kucharik et al., 2010; Zhao & Schwartz, 2003). Further, the overall average shift of about 4–5 days to earlier egg‐laying in Cooper's Hawks is similar to an average advancement of about 6–7 days for spring in Wisconsin during our study years (Kucharik et al., 2010; Serbin & Kucharik, 2009). Similarly, Stout (2008) noted a shift to earlier egg‐laying dates of about 7 days for great horned owls (*Bubo virginianus*) during 2002–2008 versus the 1970s in southeastern Wisconsin, and as with our study, the nesting period did not lengthen.

It is possible that nonclimatic factors could cause a shift in the egg‐laying dates we documented for Wisconsin Cooper's Hawks. For example, body size in several raptor species, including Cooper's Hawks, is inversely related to nesting phenology, such that larger birds tend to nest seasonally earlier and, and as aforementioned, exhibit greater productivity (Newton, 1979; Rosenfield & Bielefeldt, 1999; Warkentin, Espie, Lieski, & James, 2016). Given that size is heritable in the Cooper's Hawk, it is possible that the nesting season would advance over time as a consequence of an evolution toward larger body size in breeding birds that breed earlier (as an adaptive response) given the strong seasonal decline in reproductive output and the heritability via body size of nesting phenology (van Noordwijk et al., 1981; Rosenfield & Bielefeldt, 1999; Van der Jeugd & McCleery, 2002). However, we have shown that body size for both male and female breeding Cooper's Hawks has not changed during our study years (Rosenfield et al., 2013; Rosenfield, Bielefeldt et al., 2016). We previously linked body size in north‐central, North American breeding populations of Cooper's Hawks, including Wisconsin birds, to a phenotypically plastic response to prey size (i.e., size of hawks track size of prey; Rosenfield et al., 2010; Sonsthagen et al., 2012). We note however that the diet of breeding Cooper's Hawks in Wisconsin has not changed over our study years (Bielefeldt et al., 1992; R.N. Rosenfield, unpubl. data). Further, long‐term high survivorship and relatively high nesting densities (Rosenfield et al., 2009, 2013; Rosenfield, Hardin et al., 2016; R.N. Rosenfield, unpubl. data), along with the long‐term and relatively high average productivity indices presented herein, suggest that possible density‐dependent factors such as varying food availability and disease (investigated by Stout & Rosenfield, 2010; Rosenfield et al., 2002; Stout et al., 2005) in Wisconsin Cooper's Hawks have unlikely influenced the reproductive ecology and productivity we document here (Bielefeldt et al., 1998; Rosenfield, Bielefeldt et al., 2016). We reiterate that we have been unable to show in our multidecadal studies that habitat (i.e., rural or urban, conifer plantation or nonplantation, presumptive site quality as indexed by consistency of nesting area use, and high breeding density) is related to size of clutch or brood counts, nesting phenology, annual adult survival, and production of recruits, or fitness in Cooper's Hawks in Wisconsin (Rosenfield & Bielefeldt, 1999; Rosenfield et al., 2000, 2009; Rosenfield, Stout et al., 2015; Rosenfield, Bielefeldt et al., 2016, R.N. Rosenfield, unpubl. data). We conclude that recent climate change thus seems a plausible causative agent to the shift in egg‐laying we documented for Cooper's Hawks in Wisconsin.

The extent of the shift to earlier egg‐hatching, about 4–5 days across 36 years by Cooper's Hawks in Wisconsin, is similar to the shift of earlier egg‐hatching of about 6 days for both a similar extent of study years and a similar calendric timing for a northern European population of the congeneric and ecologically similar avivore, the Eurasian Sparrowhawk in Finland during 1973–2007 (Lehikoinen et al. 2010). Unlike Lehikoinen et al. (2010) who documented a significant temporal increase in mean brood sizes (they did not report clutch counts), we found a stable trend in mean clutch and brood sizes across our study years despite an earlier nesting schedule. While they suggested that increased average brood size was a possible response to climate change, we detected no change in average clutch and brood sizes in our study population of Cooper's Hawks during the years when climate warming was occurring in Wisconsin. We suggest that timing of food availability by perhaps earlier arriving migratory passerines in Wisconsin (especially the American Robin (Bradley et al., 1999), which is a common prey item of Cooper's Hawks in spring) is favorable for breeding when females are acquiring resources across weeks before laying eggs. We note that Snyder and Wiley (1976) found that egg‐laying by Cooper's Hawks in Arizona was triggered by the arrival of migrant songbirds, and Millsap, Breen, and Phillips (2013) also reported seasonal shifts in availability of birds may be an important trigger for nesting in Cooper's Hawks in north Florida. Our personal observations suggest that the Eastern Chipmunk (*Tamias striatus*) also may be, as it is during the nestling stage (Bielefeldt et al., 1992), important prey during the preincubation period in Wisconsin because in all years, we frequently detect their remains near nests before incubation.

It is conceivable that Cooper's Hawks could on average produce larger clutches and hence larger brood sizes on our study areas as a response to recent climate change. However, we believe this scenario as unlikely. For example, a nest with eight eggs reported by Stout (2009) for a Milwaukee, Wisconsin population of Cooper's Hawks is apparently the largest clutch size known for the species. Stout (2009) suggested failure of this nest due to the inability of the female to cover and incubate this large clutch (we note that none of the eight eggs hatched). A study in Ontario, Canada, recorded two clutches of seven eggs (Peck & James, 1983). However, these larger clutch counts appear to be anomalies (Rosenfield & Bielefeldt, 1993). We are unaware of any studies documenting more than six nestlings at one Cooper's Hawk nest. The largest clutch and brood sizes on our study areas were six eggs and six young, which maxima occurred throughout our study years but rarely (3% and 0.9% of 613 and 740 clutches and broods, respectively) and without any temporal trend (R.N. Rosenfield, unpubl. data). A clutch size of six eggs occurred at least once in each of all six generations, while a brood size of six young occurred at least once in each of the first three and in each of the last two generations but not in the fourth generation. Reproductive indices from much shorter term studies on Cooper's Hawks (most investigations of nesting Cooper's Hawks have been <6 years (Rosenfield et al., 2013)) may not be tenably and directly comparable to our long‐term findings across 36 years. Further, it is tenuous to compare reproductive indices from our work with those from studies that that did not principally locate their nests (as did we) before eggs were laid; as such, latter studies likely have calculated reproductive indices that were biased toward successful nests, whereas our productivity findings were not (Rosenfield et al., 2000). Irrespective of these possible methodological shortcomings, we note that the overall average of 4.3 eggs per clutch for our study population is exceeded by only one study (4.4 for a 5‐year study in British Columbia (Rosenfield et al., 2007)), while our overall average of 3.7 young per nest is higher than any other average brood count reported for any other Cooper's Hawk population in North America (Rosenfield & Bielefeldt, 1993; Rosenfield, Bielefeldt, et al., 2007). It is possible that adult hawks in our study years on average exhibited their maximum eco‐physiological output of eggs and young per nest given the interannual variation in environmental attributes on our apparently high breeding quality study areas (Bielefeldt et al., 1998; Rosenfield, Hardin et al., 2016), the intrinsic behavioral, physiological, and genetic qualities of breeding Cooper's Hawks on our study sites, and noting that average productivity indices were stable across six consecutive generations when egg‐hatching dates became earlier.

Adaptive responses to recent climate change in many migratory birds, which generally are believed to occur mechanistically through phenotypic plasticity in long‐lived species such as raptors (Ewert et al., 2015; but see Van Buskirk, Mulvihill, & Leberman, 2012), could of course be manifest in ecological currencies other than the productivity metrics we addressed. For example, it is possible that Cooper's Hawks in Wisconsin advanced their timing of nesting in part for social reasons regarding territoriality. An advanced nesting schedule may enhance the ability of an individual to secure and defend a territory as early as environmental conditions (e.g., food availability) permit, which behavior may enhance an occupants' ability to prevent territorial takeover concomitant with an earlier attainment of reproductive condition. An earlier seasonal initiation of nesting provides more opportunity to renest should a first clutch fail. Earlier nesting also might enhance survival of juveniles and provide a longer period of time for them to develop foraging and flight skills before the onset of migration. We note that the duration of the nesting period did not increase for Cooper's Hawks and its whole entirety advanced in timing across all study years which would consequentially lengthen the time between fledging and migration in the Great Lakes region where the onset of fall has been delayed (Ewert et al., 2015). We were unable to address these hypotheses or alternative explanations with our study design. We urge raptor researchers with long‐term reproductive data sets, especially those with cross‐generational data sets, to analyze their data within the context of how climate change potentially has (or has not) stressed various life histories in their study subjects and thus allow for elucidation of the ability (or lack thereof) of secondary avian consumers to respond to climate change and maintain or possibly enhance individual fitness and/or population viability (Ewert et al., 2015).

We highlight that our long‐term, cross‐generational stable high nesting densities, relatively high productivity indices, marked flexibility in habitat use, and high annual adult survivorship for both breeding male and female Cooper's Hawks on our study areas indicate a healthy breeding population of Cooper's Hawks (e.g., Rosenfield et al., 1995, 2009; Rosenfield, Stout et al., 2015; Rosenfield, Hardin et al., 2016; Rosenfield, Bielefeldt et al., 2016; R.N. Rosenfield, unpubl. data). Our study population appears to show resilience to and does not appear to be adversely influenced by the recent rate of changing climate. This demographic synopsis seems to favorably counter the concern regarding the aforementioned possible ill effects of an earlier migration of Wisconsin Cooper's Hawks due to recent climate warming in the Great Lakes region expressed by Sullivan et al. (2016). However, the rate of climate change is expected to increase in the Great Lakes region, and thus, further monitoring of our study population is requisite for further insights into the potential influence of a changing climate on the life histories of breeding Cooper's Hawks in Wisconsin (Ewert et al., 2015). We emphasize that such monitoring cannot be predicated on indefinite extrapolation of historical data and that reliable insights into future population status and viability during a changing climate will require periodic updates on the same ilks of demographic information assessed in earlier baselines (sensu York, Dowsley, Cornwell, Kuc, & Taylor, 2016).

## Conflict of Interest

None declared.
